# Beyond Aroma: Analytical Challenges, Metabolism and Biological Significance of Volatile Sulfur Compounds in Tomato Plants

**DOI:** 10.3390/ijms27146482

**Published:** 2026-07-21

**Authors:** Justyna Nawrocka, Kamil Szymczak, Urszula Świercz-Pietrasiak, Radosław Bonikowski

**Affiliations:** 1 Department of Plant Physiology and Biochemistry, Faculty of Biology and Environmental Protection, University of Lodz, Banacha 12/16, 90-237 Lodz, Poland; justyna.nawrocka@biol.uni.lodz.pl (J.N.); urszula.swiercz.pietrasiak@biol.uni.lodz.pl (U.Ś.-P.); 2 Institute of Natural Products and Cosmetics, Faculty of Biotechnology and Food Sciences, Lodz University of Technology, Stefanowskiego 2/22, 90-537 Lodz, Poland; radoslaw.bonikowski@p.lodz.pl

**Keywords:** volatile sulfur compounds, tomato, sulfur metabolism, plant stress responses, gas chromatography–mass spectrometry

## Abstract

Volatile sulfur compounds (VSCs) are a subgroup of plant volatile organic compounds (VOCs) that are chemically reactive and sensory-active. In tomato (*Solanum lycopersicum*), sulfur-containing volatiles such as methanethiol, dimethyl sulfide, dimethyl disulfide, dimethyl trisulfide, volatile thiols, and heterocyclic sulfur compounds are of particular relevance due to their extremely low odor thresholds and strong influence on aroma perception. Beyond their sensory importance, VSCs have been shown to be involved in plant defense mechanisms, stress responses, redox homeostasis, and multitrophic interactions with microorganisms, herbivores, and neighboring plants. The formation of these structures is governed by complex interactions between sulfur assimilation pathways, lipid peroxidation processes, environmental factors, and both enzymatic and non-enzymatic reactions. The accurate characterization of VSCs remains challenging due to the following factors: their high reactivity; their low abundance; their chemical instability; and their susceptibility to oxidation. Despite the significant enhancement in detection of VSC capabilities brought about by modern analytical approaches, such as gas chromatography coupled with sulfur-selective detection and high-resolution mass spectrometry, significant methodological limitations remain. This review summarizes the current state of knowledge on the analytical challenges of VSCs in tomato plants, their biosynthesis and biological functions, mainly in the context of plant defense.

## 1. Introduction

Among all the volatile organic compounds (VOCs) found in plants, sulfur-containing compounds, including volatile sulfur compounds (VSCs), constitute a distinct and important group. While they are produced extensively in the industrial processing of plant biomass (e.g., lignin and proteins) and need to be removed using new, modern techniques [1], in the natural life cycle of plants, they play a multifaceted physiological role [2]. Additionally, sulfur metabolism is tightly linked to redox homeostasis and stress responses, meaning that VSC emission can reflect the plant’s physiological state or environmental pressures [3,4]. Many VSCs originate from the enzymatic degradation of sulfur-containing amino acids, such as cysteine (Cys) and methionine (Met), yielding compounds with pronounced biological activity. These compounds can act as direct defense agents against herbivores and pathogens due to their toxicity or deterrent odor, and they may also function as semiochemicals, mediating plant–plant, plant–insect or even plant–pathogen interactions [2].

Sulfur and VSCs are currently receiving a lot of attention in agriculture and plant protection against diseases. The use of sulfur in various preparations is well known, and its role in alternative plant protection is becoming increasingly recognized. VSCs belong to the group of low-molecular-weight sulfur-containing chemical defense compounds (SDCs) that are thought to be associated with disease resistance, and which are vital for the survival of plants under biotic and abiotic stress conditions [5]. The effects of exogenous VSCs applied at different concentrations and in various combinations are being investigated in diverse crop plants and their surrounding environment, including associated microorganisms. VSCs are also investigated strongly in terms of agricultural activities such as harvesting, fertilization, irrigation, and pesticide application [6]. Recently, thanks to the development of new technologies, more and more studies have been undertaken on the emissions of endogenously produced VSCs by plants exposed to different factors [7,8]. For consumers, VSCs are critical contributors to flavor and aroma, even in minimal concentrations. They can impart desirable notes associated with freshness and complexity, but also off-flavors when present in excess or formed during degradation. Therefore, understanding sulfur volatiles is essential not only for elucidating plant biology but also for improving crop quality, storage, and sensory acceptance [9,10].

From an analytical and research standpoint, sulfur volatiles are of particular interest because they occur at trace levels yet have extremely low odor thresholds, making them disproportionately influential in the overall volatilomic profile. As such, they serve as sensitive markers in metabolomic studies, cultivar differentiation, and investigations of postharvest changes or processing effects [11]. Beyond analytical determination, current research on VSCs increasingly integrates computational approaches to characterize their intrinsic chemical reactivity, enabling a more comprehensive understanding of the behavior of VSCs, their occurrence, transformation, and biological activity in complex volatilomes [12]. This holistic approach to VSCs highlights their importance and demonstrates how further advancements in detection methods can shed light on their diverse roles in plants, including tomatoes.

Tomato plants (*Solanum lycopersicum* L.) are among the crop plants characterized by the emission of various volatile compounds, including potential VSCs, which have been relatively well studied in tomato products. However, despite the strong predisposition of tomato plants to research into VSC emission, the role these compounds play in protecting tomato plants from stress factors has not yet been fully elucidated [13,14]. Therefore, this review emphasizes the importance of developing various techniques for detecting VSCs produced by tomato and other plants, which will enable us to gain a clearer understanding of their generation and emission. We also summarize existing knowledge on VSCs identified in tomato plants, the sources of their synthesis that have been investigated and those that are potential, and their roles in defense in tomato plants. Within the article we try to outline the limitations and challenges associated with obtaining and interpreting data on the presence and novel functions of these compounds, and predict future research directions regarding VSCs in plants, specifically tomatoes, based on existing findings.

## 2. Analytical Methods for the Determination of VSCs

**Figure 1:**
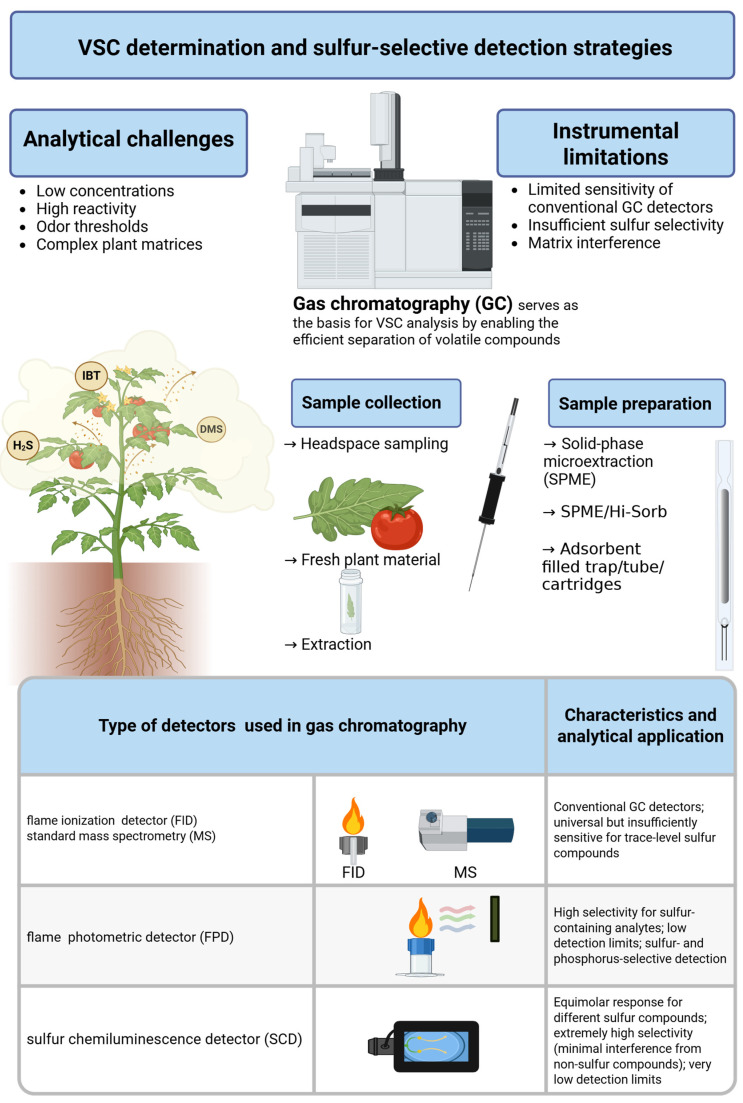
The figure presents a schematic overview of the analytical workflow and sulfur-selective detection strategies for determining VSCs in plant material. It highlights the major analytical challenges, sample collection and preparation, instrumental limitations, and the characteristics of the most commonly used gas chromatography detectors. Abbreviations: DMS—dimethyl sulfide; IBT—2-isobutylthiazole. Created in BioRender. Świercz-Pietrasiak, U. (2026) https://BioRender.com/s88ntt9 (accessed on 13 July 2026).

The determination of VSCs in plant matrices remains analytically challenging due to their low concentrations, high reactivity, and often extremely low odor thresholds. Conventional detectors used in gas chromatography (GC), such as flame ionization detectors (FIDs), or standard mass spectrometry (MS) may lack either the selectivity or sensitivity required for reliable VSC analysis. Therefore, sulfur-selective detectors—most notably the flame photometric detector (FPD) and the sulfur chemiluminescence detector (SCD)—are widely employed [15] (Figure 1).

### 2.1. Pulsed Flame Photometric Detector (PFPD)

The pulsed flame photometric detector is an advanced version of the classical FPD, specifically designed to enhance sensitivity and selectivity for heteroatoms such as sulfur and phosphorus. In PFPD, analytes eluting from the GC column are introduced into a hydrogen-rich flame, where sulfur-containing compounds undergo combustion to form excited-state sulfur species, primarily S_2_^*^ [16]. As these species return to the ground state, they emit light at characteristic wavelengths (typically around 394 nm for sulfur). What distinguishes PFPD from conventional FPD is the pulsed nature of the flame. The combustion process is temporally resolved, allowing detection of light emission in discrete time windows. Sulfur compounds exhibit longer-lived emission compared to hydrocarbon background signals, enabling time-gated detection. This significantly reduces interference and enhances selectivity toward sulfur. Additionally, the detector response for sulfur is approximately proportional to the square of sulfur concentration, which can improve sensitivity for trace-level compounds, although it requires appropriate calibration strategies [16]. Overall, PFPD provides high selectivity for sulfur-containing analytes; improved signal-to-noise ratio via temporal discrimination; and relatively low detection limits (sub-ng range).

### 2.2. Sulfur Chemiluminescence Detector (SCD)

The SCD is considered one of the most selective and sensitive detectors for VSC analysis in GC systems. Its operation is based on a multi-step process involving combustion and subsequent chemiluminescent reactions. First, sulfur-containing compounds are pyrolyzed in a high-temperature hydrogen-air furnace, converting all sulfur species quantitatively into sulfur monoxide (SO). The resulting SO is then reacted with ozone in a reaction chamber, forming excited sulfur dioxide (SO_2_^*^). As SO_2_^*^ relaxes to its ground state, it emits light (chemiluminescence), which is detected by a photomultiplier tube [17]. The key analytical advantage of SCD lies in the element-specific conversion of sulfur to a single detectable species (e.g., SO), followed by a highly efficient chemiluminescent reaction. This provides an approximately equimolar response for different sulfur compounds, largely independent of their structure, as well as extremely high selectivity with minimal interference from non-sulfur compounds and very low detection limits, often in the pg range.

### 2.3. Analytical Significance

Unlike PFPD, SCD provides a linear response over a wide concentration range, which simplifies quantification and makes it particularly suitable for complex matrices with various VSC concentrations. However, both PFPD and SCD enable reliable detection of VSCs that are otherwise difficult to quantify using non-selective detectors (e.g., FID or MS). Their sulfur specificity originates from the formation of excited sulfur species with characteristic emission in PFPD and quantitative conversion to SO followed by chemiluminescence in SCD. As a result, these detectors are indispensable in volatilomic studies of plants, where sulfur compounds, despite their trace abundance, play a crucial role in aroma and biological activity [18].

Despite the clear advantages of sulfur-selective detectors such as PFPD and SCD, in practice, analytical strategies rarely focus exclusively on volatile sulfur compounds. This is standard methodology not only for tomato but for plant matrices in general, where research is typically oriented toward the entire volatilome rather than a single compound class [19]. As a result, more universal, multi-class analytical approaches are routinely employed, even though they are noticeably less selective toward sulfur species.

### 2.4. Standard Analytical Approach

The most widely used methodologies are based on sorptive extraction techniques, which enable preconcentration of a broad spectrum of volatile and semi-volatile compounds from complex matrices. Among these, headspace solid-phase microextraction (HS-SPME) has become the standard approach [15,20]. In HS-SPME, analytes partition between the sample matrix, the headspace phase above the sample, and a polymer-coated fiber, which selectively adsorbs volatiles. And the most versatile, three-phase fiber DVB/CAR/PDMS is most commonly used. After extraction, the fiber is thermally desorbed in the GC injector. This technique is solvent-free, relatively fast, and highly adaptable, making it particularly suitable for plant volatilomics [21]. However, its efficiency for VSCs depends strongly on many factors such as fiber coating, extraction time, and temperature, and some highly volatile, reactive or ultra-trace sulfur compounds may still be underestimated or unidentifiable.

In terms of detection, HS-SPME is most commonly coupled with gas chromatography–mass spectrometry (GC-MS) [11,15]. This configuration allows for broad-spectrum identification of volatile compounds based on mass spectral libraries and retention indices [22]. While GC-MS lacks element-specific selectivity for sulfur, it remains indispensable due to its versatility and structural elucidation capabilities. For more complex samples, comprehensive two-dimensional gas chromatography (GC × GC-MS) is increasingly applied [13]. This technique provides enhanced chromatographic resolution by combining two columns of differing polarity, enabling separation of co-eluting compounds and improved detection of minor constituents, including VSCs present at trace levels [19].

Other sorptive techniques, such as stir bar sorptive extraction (SBSE) [23] or dynamic headspace (DHS) [24] methods, are also used, particularly when higher sensitivity or larger sorption capacity is required. Techniques similar to HS-SPME, such as Arrow-SPME [25] or HiSorb^TM^ [26], are also increasingly used. These approaches can offer improved recovery of certain sulfur compounds but potentially are more labor-intensive, not so popular and less routinely applied than HS-SPME.

In summary, although sulfur-selective detectors provide superior sensitivity and specificity for VSC analysis, the prevailing trend in plant studies favors comprehensive, untargeted methodologies. Techniques such as HS-SPME coupled with GC-MS or GC × GC-MS represent a compromise between analytical breadth and sensitivity, enabling simultaneous characterization of diverse volatile classes while still capturing, at least in low level, the sulfur-containing compound fraction [20].

## 3. VSCs as an Underexplored Fraction of the Plant Volatilome

The consequence of this broadly untargeted analytical approach is a substantial inconsistency in the identification of volatile sulfur compounds across studies (Table 1). Although some studies report over 1.5 thousand identified VOCs in tomato plants, only several dozen VSCs have been reported [27]. However, in the majority of routine volatilomic studies, either no sulfur compounds are reported or only one or a few are identified (Table 1). This discrepancy does not necessarily reflect their true absence, but rather the limitations of non-selective extraction and detection techniques, as well as the lack of methodological optimization specifically for sulfur compounds.

This variation appears to be driven primarily by differences in analytical selectivity rather than by the biological absence or presence of VSCs in tomato samples. Regardless of the analyzed plant material or extraction approach, most studies on tomato volatiles are still based mainly on GC-MS or GC-MS/FID methodologies, whereas sulfur-selective detectors are rarely applied. As shown in Table 1, studies based on standard GC-MS workflows usually reported only one or two VSCs, and in many cases no sulfur-containing volatiles were detected at all. In contrast, the study employing GC-PFPD in combination with GC-MS identified seven VSCs, illustrating the advantage of sulfur-selective detection for this compound class [18].

An apparent exception is the study in which more than 1500 VOCs, including 58 VSCs, were reported using GC-MS and GC × GC-ToF-MS [27]. This study was included for completeness and transparency of the review. However, such high numbers should be interpreted with caution. Comprehensive untargeted volatilomics is extremely powerful for broad chemical screening and hypothesis generation, but it does not necessarily provide the same level of confidence for VSC annotation and quantification as targeted sulfur-oriented methods. In particular, library-based identification of trace-level sulfur compounds in complex chromatograms may be affected by co-elution, low spectral quality, the presence of structurally similar compounds, and the lack of sulfur-specific confirmation. Moreover, untargeted GC-MS approaches usually do not include compound-specific calibration, recovery evaluation, or stability control for reactive sulfur species. Therefore, although such studies indicate that the sulfur fraction of the tomato volatilome may be broader than previously assumed, they should not be treated as definitive evidence of reliable VSC characterization without additional confirmation using sulfur-selective detection, authentic standards, retention indices, and targeted validation.

In addition to detector type, other factors may also contribute to the observed variation, including extraction method, sorbent selectivity, sample preparation, desorption conditions, tomato cultivar, tissue type, developmental stage, and stress or postharvest status. For example, HS-SPME, Arrow-SPME, HiSorb^TM^, Tenax/Porapak traps, and other enrichment strategies differ in sorption capacity and affinity toward low-molecular-weight, highly volatile, polar, or thermolabile sulfur compounds. Similarly, fruit, leaves, roots, and whole-plant emissions may differ substantially in precursor availability and enzymatic activity related to sulfur metabolism. Consequently, the number of detected VSCs reflects a combination of true biological variability and strong methodological bias. This further supports the need for dedicated, sulfur-focused analytical workflows rather than relying exclusively on general untargeted VOC profiling [25,26,28,29,30,31,32].

**Table 1:** Studies on volatilome (VOCs) in *S. lycopersicum* L. plant.

Extraction Method	Identification Method	No. of VOCs Identified	No. of VSCs Identified	Literature
HS-SPME	GC-MS and GC × GC-ToF-MS	1544	58	[27]
HS-SPME	GC-PFPD and GC-MS	50	7	[18]
HS-ArrowSPME	GC × GC-Q/ToF-MS	227	2	[25]
HS-SPME	GC-MS	55	2	[33]
HS-SPME	GC × GC-ToF-MS	100	1	[13]
PDMS tubes and HiSorb probes	GC-Q/ToF-MS	17	1	[26]
HS-SPME	GC-MS	36	1	[34]
Tenax adsorbent trap	GC-MS	29	1	[28]
HS-SPME	GC × GC-ToF-MS	24	0	[35]
Tenax TA tubes	GC-MS	33	0	[29]
Porapak Q tubes	GC-MS	55	0	[30]
HS-SPME	GC-MS	18	0	[36]
Porapak Q cartridges	GC-MS	84	0	[31]
Super-Q adsorbent	GC-MS	25	0	[37]
Tenax TA and Carbograph 5TD cartridges	GC-MS/FID	29	0	[32]

At the same time, the strong focus on the overall volatilome means that sulfur compounds remain significantly undercharacterized. Only a limited number of studies have deliberately targeted VSCs using selective detectors or tailored extraction protocols [18]. As a result, knowledge regarding their distribution across plant tissues, their biosynthetic pathways, and their variability between cultivars or environmental conditions is still relatively sparse.

Another important limitation in the analysis of VSCs is the thermolability of many sulfur-containing compounds, which has direct implications for the most commonly applied extraction techniques. In standard HS-SPME workflows, analytes are thermally desorbed from the fiber in the GC injector, typically at temperatures in the range of 200–250 °C. While this ensures efficient transfer of most volatile compounds onto the column at a time, such conditions can be excessively harsh for many sulfur-containing molecules [38].

A number of VSCs—particularly thiols, thioesters, and certain polysulfides—are prone to thermal degradation, rearrangement, or oxidation under these conditions. As a consequence, the original compounds may be partially or completely decomposed during desorption, leading to the formation of secondary artifacts (e.g., sulfides derived from thiols) or, conversely, to complete signal loss. This not only compromises accurate identification but may also distort the apparent composition of the sulfur fraction, contributing to the inconsistencies observed across studies. In this context, alternative desorption strategies offer a significant advantage. Techniques based on thermal desorption units (TDUs) [28] allow for controlled, gradual heating of the sample or sorbent. Instead of instantaneous exposure to high temperatures, analytes are released under lower initial temperatures followed by programmed temperature ramps, which reduces the risk of thermal degradation. Additionally, such systems can be coupled with cryogenic focusing (e.g., cold injection systems), further improving analyte transfer efficiency and peak shape while preserving thermally labile compounds [26,29]. As a result, TDU-based approaches or other low-temperature desorption techniques provide a more suitable analytical framework for VSC determination. They enable improved recovery of reactive and unstable sulfur species, offering a more representative profile of the plant volatilome. This is particularly important for compounds that are not only present at trace levels but are also highly sensitive to analytical conditions.

The analytical gap in VSC screening is particularly important given that VSCs often exhibit extremely low odor thresholds, meaning that even trace concentrations can have a disproportionately large impact on aroma perception. Consequently, compounds that are barely detectable analytically may still be key contributors to sensory quality. From an evolutionary perspective, such compounds may play critical roles in plant defense, signaling, or ecological interactions. From a consumer standpoint, they may significantly influence flavor acceptance and perceived freshness. Therefore, despite their low abundance and analytical challenges, VSCs likely represent a functionally important but still insufficiently explored fraction of the plant volatilome.

## 4. Classification and Chemical Diversity of VSCs in Tomato

VSCs identified in tomato represent a chemically diverse group of molecules that differ significantly in structure, reactivity, and sensory impact. These compounds can be broadly classified into several functional groups, including sulfides, thiols, thioesters, and sulfur-containing heterocycles (mainly thiazoles). Each of these classes originates from distinct biochemical pathways, most commonly linked to the degradation of sulfur-containing amino acids such as Cys and Met, or to thermally induced reactions occurring during processing (in the case of tomato fruit as processed for food) [11].

The structural diversity of VSCs is directly reflected in their physicochemical properties, such as volatility, stability, and odor activity. Notably, even minor structural differences—such as the number of sulfur atoms or the presence of additional functional groups—can result in substantial changes in sensory perception [12]. Therefore, classification of these compounds is not only chemically justified but also essential for understanding their role in shaping the overall volatilome of tomato.

### 4.1. Sulfides

Sulfides constitute one of the primary classes of volatile sulfur compounds identified in tomato, encompassing mono-, di-, and trisulfides with the general structures R–S–R′, R–S–S–R′, and R–S–S–S–R′, respectively. Among the most commonly reported representatives are dimethyl sulfide (DMS), dimethyl disulfide (DMDS), and dimethyl trisulfide (DMTS), which differ in the number of sulfur atoms bridging the organic moieties. This structural variation has a pronounced effect on their chemical stability, volatility, and sensory characteristics. From a biochemical perspective, sulfides are mainly formed through the degradation of sulfur-containing amino acids, particularly Met [11]. Enzymatic cleavage and subsequent transformations (e.g., via Met γ-lyase activity) can yield methanethiol, which is a key intermediate [39]. Methanethiol is highly reactive and readily undergoes oxidation and condensation reactions, leading to the formation of di- and trisulfides. These processes may occur both enzymatically and non-enzymatically, and can be further influenced by factors such as tissue disruption, oxidative conditions, and temperature [40].

In terms of occurrence, sulfides are typically detected in both fresh and processed tomato matrices, although their concentrations and relative proportions vary depending on ripeness, cultivar, and sample treatment. Thermal processing, in particular, tends to promote the formation of higher polysulfides, such as DMTS, due to enhanced degradation of precursors and increased reaction rates [10,41].

In sensory terms, sulfides are characterized by intense odor properties, often described as cabbage-like, onion-like, or cooked vegetable notes [11]. While these attributes may be undesirable at high concentrations, at trace levels they contribute to the complexity and authenticity of the tomato aroma. Notably, dimethyl trisulfide has an extremely low odor threshold and is considered a potent aroma-active compound despite typically occurring at very low concentrations [10].

From an analytical standpoint, sulfides are generally more stable and easier to detect than thiols, which makes them the most frequently reported in GC-MS-based volatilomic studies. However, their accurate quantification still benefits from sulfur-selective detection due to potential co-elution and matrix interferences. As such, sulfides represent a relatively well-documented yet still not fully characterized subgroup of VSCs in tomato.

### 4.2. Thiols

Thiols (also named mercaptans) represent a particularly important and analytically challenging class of volatile sulfur compounds in tomato, characterized by the presence of the sulfhydryl functional group (–SH). Structurally, they are typically described by the general formula R–SH and include both low-molecular-weight compounds, such as methanethiol and ethanethiol, and more structurally complex molecules like furanthiols. Despite often occurring at extremely low concentrations, thiols are among the most potent odor-active compounds due to their exceptionally low sensory thresholds [10].

Biochemically, thiols are primarily derived from the degradation of sulfur-containing amino acids, especially Cys and Met. Enzymatic pathways involving C–S lyases can directly generate thiols such as methanethiol, which serves as a key intermediate in sulfur metabolism [11]. Additionally, thiols may be formed through secondary transformations, including reactions involving lipid oxidation products or Maillard-type chemistry, particularly under conditions of tissue disruption or thermal processing for food. Their formation is therefore closely linked to both enzymatic activity and physicochemical conditions within the plant matrix.

A defining feature of thiols is their high chemical reactivity. The –SH group is prone to oxidation, readily forming disulfides, which makes thiols inherently unstable during sampling, extraction, and analysis. This reactivity significantly complicates their detection and often leads to their underrepresentation in standard GC-MS-based studies. As a result, dedicated analytical strategies—such as derivatization, careful control of extraction conditions, or the use of sulfur-selective detectors—are required to reliably capture their presence.

From a sensory perspective, thiols contribute intensely to aroma profiles, typically imparting notes described as garlic-like, onion-like, roasted, or even coffee-like, depending on their structure. Certain thiols, such as 2-methyl-3-furanthiol, are recognized as key aroma-active compounds in thermally processed products and their presence in fresh fruit may be related to too harsh analysis conditions. Importantly, due to their extremely low odor thresholds, even trace amounts can have a disproportionate impact on overall flavor perception [10,42].

In the context of tomato volatilomics, thiols remain one of the least comprehensively characterized groups of VSCs. Their low abundance, high reactivity, and analytical instability mean that they are often overlooked in untargeted studies. Nevertheless, their central role in sulfur metabolism suggests that they constitute a critical, albeit underexplored, component of the tomato volatilome.

### 4.3. Thiazoles

Sulfur-containing heterocycles, particularly thiazoles, represent a distinct group of volatile sulfur compounds in tomato characterized by the incorporation of sulfur within an aromatic ring structure. Thiazoles are five-membered heterocycles containing both sulfur and nitrogen atoms, which confer specific chemical stability and unique sensory properties compared to aliphatic sulfur compounds such as thiols or sulfides. Commonly reported representatives in tomato include compounds such as 2-isobutylthiazole, 2-secbutylthiazole, 2-propylthiazole and related alkyl-substituted derivatives [18].

In contrast to thiols and sulfides, which are primarily formed via enzymatic degradation of sulfur-containing amino acids, thiazoles are typically associated with more complex formation pathways. Their generation often involves reactions between sulfur-containing precursors (e.g., Cys or its degradation products) and carbonyl compounds, including intermediates derived from carbohydrate metabolism [11]. These reactions may occur enzymatically in fresh plant tissue but are also strongly linked to non-enzymatic processes such as Maillard-type reactions, particularly during thermal treatment or tissue disruption [42].

From a sensory perspective, thiazoles are generally associated with green, earthy, roasted, or nutty notes. In fresh tomato, certain thiazoles—especially 2-isobutylthiazole—are considered key contributors to the characteristic “green” and slightly pungent aroma of unripe fruits [9]. Unlike highly reactive thiols, thiazoles exhibit greater chemical stability, which facilitates their detection in routine GC-MS analyses and explains why they are more consistently reported across volatilomic studies.

Despite being more analytically accessible than other VSC classes, sulfur-containing heterocycles still represent a relatively small fraction of the overall tomato volatilome. However, their structural complexity, distinct formation pathways, and notable sensory contributions make them an important link between primary sulfur metabolism and the development of characteristic aroma profiles in both fresh and processed tomato plant and products.

### 4.4. Thiophenes

Thiophenes are another class of sulfur-containing heterocyclic compounds detected in tomato, structurally distinct from thiazoles by the absence of nitrogen in the ring. They consist of a five-membered aromatic ring incorporating a sulfur atom and can occur in simple unsubstituted forms or as alkyl- and oxygen-substituted derivatives [43]. Although typically present at trace levels, thiophenes are noteworthy due to their chemical stability, which makes them relatively detectable by standard GC-MS methods.

The formation of thiophenes in tomato is primarily associated with secondary transformations of sulfur precursors, including thiols, sulfides, and reactive carbonyl compounds. These reactions may occur enzymatically at low levels in intact plant tissue, but are more pronounced during thermal processing, tissue disruption, or oxidative conditions, which promote cyclization and aromatization reactions [11]. Consequently, thiophenes are often more prominent in cooked or processed tomato products, although some are detectable in fresh fruit under sensitive analytical conditions.

In sensory terms, thiophenes contribute roasted, nutty, or cooked vegetable notes [44], which complement the aroma profile established by other VSCs such as thiols and thiazoles. While they are less odor-active than certain thiols due to higher odor thresholds, they can still modulate overall flavor complexity.

Analytically, thiophenes benefit from relative stability and lower reactivity, reducing the risk of degradation during high-temperature desorption steps in GC [44]. However, their low natural abundance means that sensitive detection techniques, sometimes coupled with high-volume preconcentration methods and thermal desorption, are still necessary to reliably characterize their presence in tomato volatilomes. This combination of stability and low abundance positions thiophenes as a minor but functionally important class of VSCs, bridging the gap between highly reactive sulfur species and more persistent heterocyclic compounds like thiazoles.

### 4.5. Other VSCs

In addition to the major classes such as thiols, sulfides, and thiazoles, several other VSCs have been reported in tomato, although they occur less consistently and are often present at trace levels. Among these, S-methyl thioacetate is one of the few compounds that has been detected in volatilomic studies [45]. As a thioester, it represents a structurally distinct subgroup of VSCs, typically formed through reactions between thiols (e.g., methanethiol) and acyl donors. Its sensory properties are often described as sulfurous with slightly fruity or ester-like nuances, suggesting a potential modulatory role in aroma perception despite its low abundance.

Other sulfur-containing molecules, such as hydrogen sulfide (H_2_S), sulfur dioxide (SO_2_), carbon disulfide (CS_2_) and carbonyl sulfide (COS), may also be present in tomato tissues as intermediates or by-products of sulfur metabolism. These compounds are biochemically plausible, given their involvement in pathways related to Cys degradation and broader sulfur cycling within plant cells. However, their extremely high volatility and low molecular weight make them particularly difficult to capture and analyze using standard volatilomic approaches such as HS-SPME-GC-MS.

From an analytical standpoint, those small molecules exhibit high diffusivity and weak sorption to commonly used extraction phases, which lead to significant losses during sampling and preconcentration. Additionally, their detection often requires specialized instrumentation (e.g., gas-specific detectors, online sampling systems, or cryogenic trapping) and carefully controlled conditions to prevent volatilization losses. As a result, these compounds are rarely reported in routine studies, and their presence in tomato volatilomes is rather considered than reliably confirmed [27,39,41].

Overall, this group of other VSCs, but also thiols, highlights an important limitation of current analytical workflows: compounds that are either highly reactive, thermolabile or extremely volatile remain difficult to assess, despite their potential biochemical relevance and sensory influence (Table 2).

## 5. Metabolism and Biological Significance of VSCs in Tomato

The constitutive emission and perception of VOCs play unappreciated housekeeping roles in mediating intra-plant, inter-plant, plant–animal, and plant–microorganism communication [47,48]. The chemical composition and emission of VOCs by plants are species-, organ- and tissue-specific; depend on the physical (e.g., leaf area) and physiological properties (e.g., metabolic activity) of plants; and are regulated by environmental factors such as light intensity, atmospheric chemistry, temperature, relative humidity, soil fertility, wounding, or pathogen challenge [48,49,50]. Among all VOCs released by plants, alongside terpenoids, fatty acid derivatives (GLVs), and aromatic compounds which constitute the majority [14], increasing attention is being paid to minor compound groups such as volatile nitrogen-containing compounds and VSCs, whose role in plant functioning has only recently been recognized, and there are still many unanswered questions about the regulation of VSC metabolism and their role in plant functioning [51,52].

Tomato plants are an important species for the analysis of VOCs, including VSCs, in the context of plant functioning and response to environmental and stress factors, as well as practical consumer interest [53,54]. In terms of the latter, consumers have increasingly complained about the poor flavor of modern tomatoes over the last few decades. In response, breeders, agronomists and food technologists have started paying more attention to this trait, resulting in a constant increase in the number of Scopus papers dealing with VOCs in tomatoes over the last 30 years [54]. Most of the VOCs identified in tomato plants are classified according to their biosynthetic origin, as VOCs derived from (i) fatty acids, (ii) sulfur and branched-chain amino acids, (iii) carotenoid catabolism, and (iv) phenolics and phenylpropanoids [53]. Despite the growing number of studies on the participation of VSCs in the volatilome of tomato plants, many of these studies have focused on fruit, including processed fruit. So far, few studies have examined how VSCs and changes in their metabolism are involved in tomato plant responses to environmental factors [54,55]. This area remains largely unexplored, and detailed future research using new methods of analysis is essential to clarify changes in the metabolism and emission of different VOCs, including VSCs, by crop plants such as tomatoes growing under the influence of various factors.

### 5.1. Sulfur and VSC Metabolism in Plants, Including Tomato

**Figure 2:**
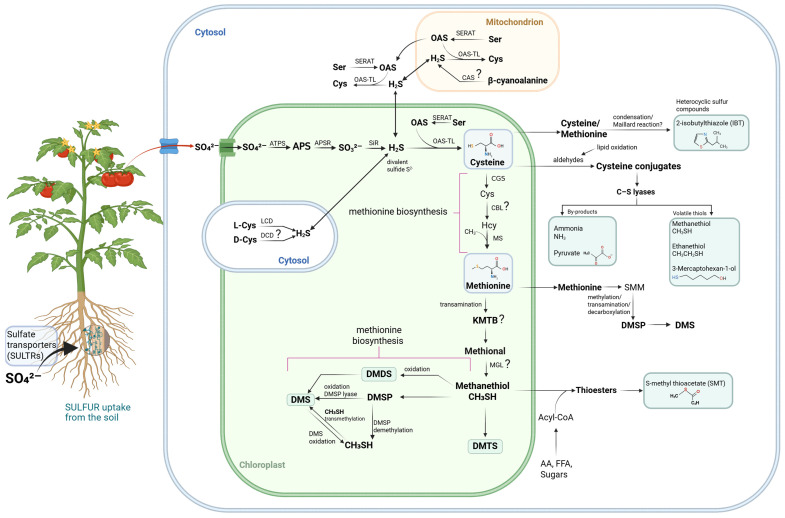
The figure illustrates the major pathways of sulfur metabolism leading to the formation of VSCs in plants, including documented and possible (?) steps in tomato plants. Abbreviations: SULTRs—sulfate transporters; APS—adenosine-5′-phosphosulfate; ATPS—adenosine triphosphate sulfurylase; APSR—APS reductase; SiR—sulfite reductase; OAS—O-acetylserine; SERAT—serine acetyltransferase; OAS-TL—O-acetylserine (thiol) lyase; Ser—serine; Cys—cysteine; LCD—L-cysteine desulfhydrases; DCD—D-cysteine desulfhydrase; CAS—β-cyanoalanine synthase; CGS—cystathionine γ-synthase; CBL—cystathionine β-lyase; MS—methionine synthase; C–S lyases—carbon–sulfur lyases; SMM—S-methylmethionine; DMS—dimethyl sulfide; DMDS—dimethyl disulfide; DMSP—dimethylsulfoniopropionate; DMTS—dimethyl trisulfide; KMTB—4-methylthio-2-oxobutyrate; MGL—methionine γ-lyase; AA—amino acid; FFA—free fatty acid. Created in BioRender. Świercz-Pietrasiak, U. (2026) https://BioRender.com/sub2a3q (accessed on 13 July 2026).

The metabolism of many VSCs found in plants, including tomatoes, has not yet been examined, and many of the enzymes and genes involved remain unknown. Figure 2 presents the key identified and presumed biosynthesis pathways of VSC and other sulfur-containing compounds in tomatoes and other plants, as derived from the available literature.

The metabolism of VSCs in plants including tomato is strongly related to the assimilation of inorganic sulfur (S) being an essential nutrient for plant growth, ranked fourth after nitrogen, phosphorus, and potassium. It is a component of amino acids, vitamins, coenzymes, and other key molecules, playing an important role in photosynthesis, respiration, and cell membrane formation [56,57]. Sulfur deficiency in tomato has been demonstrated to disrupt these processes, leading to altered nutrient homeostasis; reduced levels of S-containing metabolites, including sulfides, Cys, γ-glutamyl-Cys, glutathione (GSH), and S-adenosyl-Met; and increased accumulation of serine (Ser) and O-acetylserine (OAS) in shoots and roots [57]. Sulfur assimilation and reduction is an energy-dependent process closely linked to cellular metabolism. Although sulfate uptake and reduction are generally stimulated by light, these processes may also proceed in darkness depending on the physiological state and metabolic activity of the cell [56]. Although plant leaves can absorb gaseous SO_2_ and H_2_S, the main source of S is taken up in the form of sulfate (SO_4_^2−^), which is absorbed from the soil and transported within the plant via sulfate transporters (SULTRs) [58]. Recent studies have identified 12 genes encoding SULTRs in tomato plants [59]. Sulfate uptake and transport are regulated by sulfate limitation (SLIM1), an EIN3/EIL (Ethylene Insensitive 3/Ethylene Insensitive 3-Like) family transcription factor that controls gene expression in response to sulfur deficiency [57,60,61]. The importance of the EIN3/EIL family in controlling sulfur metabolism is supported by transcriptomic (RNA-seq) analyses, which demonstrated that expression of SIEIL (EIN3/EIL homolog) genes plays an important role in the sulphate deficiency response in tomato plants [57]. This opens the way for further research into other tomato protein homologues that may be involved in a key step of sulfur uptake and subsequent metabolism. In addition to directly regulating genes responsive to sulfur, SLIM1 has been shown to induce the accumulation of microRNA-395 (miRNA395) during sulfur starvation. miRNA395 has been demonstrated to modulate sulfur homeostasis by targeting transcripts encoding ATP sulfurylase (ATPS) and SULTR proteins, thereby regulating sulphate assimilation and transport according to sulfur availability [61]. The SLIM1–miRNA395 regulatory module coordinates the uptake, transport and assimilation of sulfur, thereby demonstrating the integration of transcriptional and post-transcriptional mechanisms in the maintenance of sulfur homeostasis also in tomato plants [57].

The first committed step of sulfur assimilation in tomato, as in other higher plants, is the activation of inorganic sulfate (SO_4_^2−^) by ATPS, which catalyzes the formation of adenosine-5′-phosphosulfate (APS). Subsequent to this, APS is reduced by APS reductase (APSR) to sulfite ion (SO_3_^2−^), which is further reduced by ferredoxin-dependent sulfite reductase (SiR) to H_2_S. Hydrogen sulfide is the primary reduced sulfur source in plant cells [61,62].

Sulfide generated by SiR serves as the sulfur donor for Cys biosynthesis. Serine acetyltransferase (SERAT) catalyzes the formation of O-acetylserine (OAS) from serine and acetyl-CoA, providing the sulfide acceptor. Subsequently, O-acetylserine (thiol) lyase (OAS-TL) incorporates sulfide into OAS to produce Cys. SERAT and OAS-TL may be localized in the cytosol, plastids, and mitochondria, enabling coordinated Cys biosynthesis and sulfur fluxes across cellular compartments [61,63]. In tomato plants, SERAT was presented to catalyze the rate-limiting step of OAS formation from serine and acetyl-CoA [64], while OAS-TL was detected to catalyze the biogenesis of Cys in chloroplasts and in the cytosol [65].

Beyond its metabolic role as a precursor for Met and a central sulfur donor, Cys is tightly connected to H_2_S homeostasis and signaling processes [61,66,67]. In tomato plants the predominant source of endogenous H_2_S is L-Cys desulfhydrase (LCD) [68], while broader plant literature includes D-Cys desulfhydrase (DCD), β-cyanoalanine synthase (CAS), and SiR as additional H_2_S sources [69], but their tomato-specific contributions are not established. Nevertheless, in the Solanaceae family, OAS-TL and related enzymes appear to influence cellular H_2_S homeostasis indirectly due to the reversibility of the reaction and associated sulfur metabolism (e.g., the regulation of the balance between sulfur assimilation and desulfhydration pathways) [65].

Cys and Met act as important precursors for VSCs [70]. Met biosynthesis, studied extensively in *Arabidopsis*, showed that it might involve two consecutive steps catalyzed by cystathionine γ-synthase (CGS) and cystathionine β-lyase (CBL), producing homocysteine, with cystathionine (Cyst) as an intermediate product. Homocysteine is then methylated to Met by Met synthase (MS) using methyltetrahydrofolate as the methyl donor [70]. Models of methionine biosynthesis are currently being developed in tomato plants, incorporating elements such as CGS, CBL, Hcy and MS, primarily in the context of ripening [71]. However, further research is required to confirm the role of these enzymes and metabolites in the basic metabolism of tomato plants. Met is possibly converted via transamination to 4-methylthio-2-oxobutyrate (KMBT), which may be subsequently transformed into methional, an important aroma compound in tomato, and further methanethiol, a pivotal intermediate in the formation of simple VSCs [72]. However, further research on tomato plants would provide more clarity on this pathway.

Methanethiol is highly reactive and readily undergoes oxidation and condensation reactions, leading to the formation of di- and trisulfides [18]. These processes may occur both enzymatically and non-enzymatically. The biosynthesis of dimethylsulfoniopropionate (DMSP), a direct precursor of reactive VSCs, may proceed via several different pathways, including two methylation pathways, a transamination pathway and a decarboxylation pathway [39,56,73]. In tomato, homologs of all four genes involved in DMSP biosynthesis, methylmethionine methyltransferase (MMT), S-methylmethionine decarboxylase (SDC), DMSP-amine oxidase (DOX), and aldehyde dehydrogenase (ALDH), are present [73]. However, the tomato homolog of SDC functions as a conventional ornithine decarboxylase rather than an SDC and therefore does not catalyze the key decarboxylation step in DMSP biosynthesis. Consequently, tomato is thought to rely on alternative SDC-independent biosynthetic routes, such as the transamination pathway [73]. Although DOX, MMT, and ALDH homologs are also present, the tomato DOX enzyme exhibits substantial activity, whereas MMT and ALDH appear to perform conserved functions in primary metabolism [73]. DMSP is predominantly localized in the cytoplasm, although a substantial fraction may also occur in chloroplasts [54,74]; however, the subcellular localization of DMSP has not yet been determined in tomato. DMSP can be metabolized through two major pathways: the cleavage pathway, which generates DMS, and the demethylation pathway, which produces methanethiol. Based on the literature presenting results in other plants, we may hypothesize that in tomato, methanethiol may be related to the generation of DMDS or DMTS, or further converted through sulfur metabolic pathways [18,20,50,54,75]. Despite the fact that DMSP is synthesized predominantly through the methionine methylation pathway, the complete genetic network controlling this process remains to be elucidated [73,76,77].

Concurrently, a significant pathway pertains to the synthesis of Cys conjugates with aldehydes, which are often derived from lipid oxidation. These conjugates are then cleaved by carbon sulfur lyases (C–S lyases), releasing volatile thiols such as methanethiol, ethanethiol, and 3-mercaptohexan-1-ol, along with by-products such as ammonia and pyruvate. This pathway is considered to be a significant source of sulfur-containing aroma compounds in tomato [54,78,79]. Furthermore, heterocyclic sulfur compounds can be formed through condensation and cyclization reactions involving sulfur-containing precursors and carbonyl compounds. A notable example is 2-isobutylthiazole, a heterocyclic sulfur compound formed through condensation reactions involving amino acid-derived precursors, although its biosynthetic pathway remains incompletely resolved. This compound is one of the key components of the characteristic aroma of tomatoes [54,66]. Transcription factors SlEIL play an important role in regulating the biosynthesis of 2-isobutylthiazole, as demonstrated by Wang et al. [80]. They reported significantly lower levels of this compound in the SlEILs-CS1 and SlEILs-CS2 co-suppression lines than in wild-type fruits. However, the precise biosynthetic mechanism of this and other heterocyclic sulfur compounds remains incompletely understood and is still better known for forming through Maillard-type thermal reactions than through natural biosynthesis in plants [79].

The synthesis of VSCs is subject to stringent regulation and is contingent on factors such as the fruit ripening stage, the availability of precursors (Cys and Met), and the compartmentalization of enzymes and substrates within the cell. During the process of ripening or tissue disruption, enzymes and substrates come into contact, leading to the rapid formation and release of volatile sulfur compounds [74,81]. Despite these advances, the biosynthesis pathways of many VSCs in plants, including tomato, remains insufficiently characterized, and numerous enzymes and genes involved have yet to be identified.

### 5.2. The Role of VSCs in Plant Protection Against Environmental Stresses

**Figure 3:**
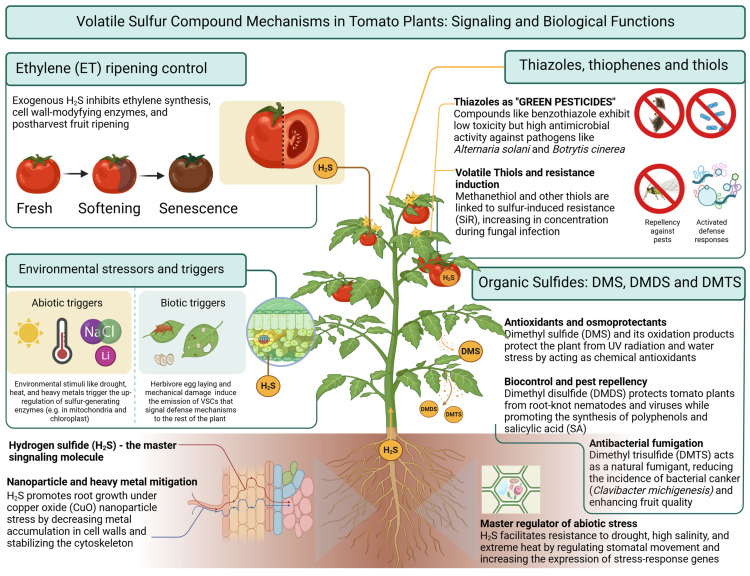
The figure summarizes the major biological functions associated with sulfur-derived metabolites in tomato plants. Sulfur metabolism gives rise to H_2_S and diverse VSCs, which play important roles in plant development, stress adaptation, defense responses, and environment interactions. Created in BioRender. Świercz-Pietrasiak, U. (2026) https://BioRender.com/beqr0tt (accessed on 13 July 2026).

Research into the role of VSCs in relation to plants is currently multifaceted. Some studies focus on the ability of soil-released VSCs to promote plant growth and biomass accumulation, and to combat pathogens directly and indirectly, in plant–microorganism interactions [82]. Furthermore, recent research has focused on the mutual interaction between microorganisms and plants with regard to sulfur metabolism, including VSCs from both the host and the microbiome. These compounds are coordinated during root colonization and influence plant resistance and growth [7]. Concurrent research on fruits and vegetables has concentrated on the role of sulfur-containing compounds, including volatiles, in determining their olfactory and gustatory characteristics [83]. Compared to the extensively researched mineral sulfur, there has been a growing focus on researching various VSCs to comprehend their function in plant processes and defense mechanisms against abiotic and biotic stress factors, including air pollution, extreme heat events, droughts, and the impact of herbivores and pathogens (Figure 3) [49,84]. Volatile sulfur-containing substances emitted by plants bearing herbivorous insect eggs are also being investigated for their role in inducing defense mechanisms in intact plants [85].

With regard to tomato plants, the primary research focuses on H_2_S [68]. Some studies focus on sulfides but more often on studies of tomato fruits and products than plants [86]. To the best of our knowledge, there is currently a significant knowledge gap concerning the impact of environmental factors on the production of volatile thiols and thiazoles in tomato plants. However, thanks to modern technology, an increasing number of plants are now being assessed for their emissions [87,88].

A growing body of research has confirmed the role of H_2_S in seed germination, root organogenesis, stomatal density and movement, inhibition of senescence, and response to drought, high salinity, heavy metal, and nanoparticle stress. However, the precise mechanisms underlying H_2_S action often remain unclear [68,89,90]. The potential mechanisms by which H_2_S regulates the physiological processes of tomato plants are investigated. For example, the activity of H_2_S-generating enzymes and the expression of genes involved in H_2_S production in tomato plants increased in response to environmental stimuli such as heat and drought stress [91]. They were also up-regulated by exogenous abscisic acid (ABA), salicylic acid (SA), and naphthaleneacetic acid (NAA), and then gibberellic acid (GA), jasmonic acid (JA), and 1-aminocyclopropane-1-carboxylate (ACC), suggesting that H_2_S functioning in tomato may interact with different molecules, including phytohormones [91]. Moreover, H_2_S has been shown to play a role in tomato reproduction, fruit development and ripening by regulating genes involved in cell wall degradation and the ethylene (ET) signaling pathway. Applying exogenous H_2_S has been shown to inhibit endogenous ET synthesis and regulate ET and reactive oxygen species (ROS) signaling in tomato fruits [92,93]. Liu et al. [93] found that H_2_S inhibited ET-induced abscission in tomato petioles through modulation of auxin content, post-translational suppression of cell wall-modifying enzyme activity, and inhibition of the expression of genes involved in abscission [93]. H_2_S was found to inhibit ET synthesis through persulfidation and inhibition of ACC oxidase activity, thereby alleviating tomato ripening and senescence [94]. In the presence of CuO nanoparticles, H_2_S has been observed to promote tomato root growth by inhibiting the accumulation and translocation of Cu, while concurrently preserving the integrity of the cell wall and maintaining the organization of the cytoskeleton [94]. At the subcellular level in chloroplasts and/or mitochondria, the role of H_2_S in Fe-S cluster formation, regulation of membrane stability and antioxidant systems, and CN^−^ detoxification is discussed [95]; however, the research on tomatoes in this area still needs to be completed. Significantly less research has been conducted on the effects of H_2_S on pathogens and herbivores, particularly in the context of tomato plants. However, there is evidence to suggest that it could protect postharvest tomato against certain pathogens, including *Aspergillus niger* and *Penicillium italicum*, by a direct negative effect on the growth, development and functioning of pathogens [96]. Although there is a lack of direct evidence, H_2_S-mediated pathogen protection may be indirectly associated with the regulation of sugar, protein, organic acid, and secondary metabolite metabolism, as suggested by studies involving the use of exogenous H_2_S in postharvest tomatoes [97]. In other plant species, it has been shown that H_2_S enhances immunity through SA signaling, PR gene induction and protein persulfidation. However, these mechanisms have yet to be investigated in tomato [98].

Methanethiol and its sulfide derivatives are increasingly recognized for their roles in plant responses to changing environmental conditions; however, in tomato, research has focused mainly on processed products, while studies on their functions in fresh fruits and plant responses to environmental stresses remain largely unexplored despite the identification of methanethiol and some sulfides in tomato plants [18,86,99]. Nevertheless, there are studies in which tomato plants were treated both with methanethiol and its derivatives, as exogenous repellents in plant protection against different pathogens and herbivores. For example, treatment of tomato plants with DMDS protected them against root-knot nematode (*Meloidogyne incognita*) infestation and tomato brown rugose fruit virus in a greenhouse [100]. The effect of DMDS on the fitness of tomato plants and its fungicidal potential against a fungal phytopathogen, *Sclerotinia minor*, were investigated by Tyagi et al. [101]. In the studies, DMDS inhibited mycelial growth, sclerotia formation, and germination, as well as promoted growth of tomato plants, reduced disease symptoms, and regulated the expression of genes related to growth, and SA induced systemic resistance (pathogenesis-related PR1 and PR5 proteins) against the pathogen in tomato [101]. Another sulfide, dimethyl trisulfide (DMTS), has been shown to have antibacterial properties. Fumigation with DMTS enhanced fruit quality and reduced the postharvest incidence of bacterial canker in tomato caused by *Clavibacter michiganensis*, by the direct inhibition of the pathogen’s growth and development, and by its influence on tomato biochemistry, including increased ascorbic acid (AA), total soluble solids, total soluble sugars contents, and acidity, and it inhibited ET biosynthesis at the molecular level [102].

As indicated by the literature on the Solanaceae family and other plants, it can be hypothesized that DMS and DMSP in tomatoes may perform various biological functions, including detoxifying excess sulfur, mitigating salt stress, providing cryoprotection and deterring herbivores [75]. Based on further literature, it can be hypothesized that methanethiol, DMS and DMDS, which are emitted following mechanical wounding of leaves, might increase the content of total polyphenolics and aromatic amino acids, as well as the emission of other VOCs, including methyl salicylate (MeSA), and the activity and expression of genes that encode enzymes involved in the synthesis of polyphenols and PR proteins. This has been observed in the management of the Asian citrus psyllid, which is the insect vector of the huanglongbing pathogen [103]. Other speculations may be related to findings in other plants that oxidation products of DMS, such as dimethyl sulfoxide and methanesulfinic acid, act as antioxidants, osmo-protectants or protectants against UV radiation and metal stress [75].

Research on thiols in plants has yielded a substantial body of knowledge regarding their biological functions. S-containing volatile thiols are more often included into the wide variety of antimicrobial or antioxidative thiol compounds such as GSH, thionins, defensins, phytoalexins, and glucosinolates constituting the primary reservoir of sulfur, which can be transformed into reactive forms under changing environmental conditions [104,105]. However, the role of volatile thiols and their derivatives in plant responses to environmental factors remains to be fully elucidated. The presence of volatile thiols, which are produced during the secondary metabolism of certain plant species, has been demonstrated to play a significant role in the development of food aroma. However, the majority of sulfur compounds present in food products do not originate directly from plant metabolism. Instead, they are formed during the fermentation process or further food processing. Thiols have been demonstrated to be responsible for both desirable aromatic notes and unpleasant odors, which are often associated with the degradation of sulfur-containing amino acids [106]. Despite the difficulties involved in detecting volatile thiols, an increasing number of scientists are conducting research into these compounds in plant materials and plants. This is because, due to their strong reactivity and volatility, these compounds can play a key role in various metabolic processes involving sulfur [88,107]. Based on the available data, it can be concluded that volatile thiols may play a significant role in the plant response to biotic stress. Sulfur-induced plant resistance (SIR) in Brassicaceae plants against harmful organisms has been shown to be associated with an increased thiol content [108]. Conversely, snail feeding was found to increase the levels of oxidized thiols, which may be directly volatile thiols or their precursors in cabbage leaves and roots, indicating the involvement of these compounds in plant defense mechanisms [109]. One of the volatile thiol compounds detected by modern techniques in the VOCs emitted by plants is the aforementioned methanethiol, previously described as one of the VOCs whose concentration increases during the overripe stage of maturation [110]. For example, thanks to the use of nanobionic sensor plants, the increase in this compound emission was detected in the volatilome released by strawberry fruits infected with fungal pathogen *Botrytis cinerea* [87].

Despite the growing evidence of their presence in tomato fruit and plants [18], the role of volatile thiazoles in tomato plant physiology and their function in protecting plants against environmental stressors remains to be elucidated. However, the significance of this role is evidenced by the recognized antipathogenic properties of compounds containing the thiazole and isothiazole structure [111]. They are employed in agriculture as green pesticides, fungicides, insecticides, and herbicides due to their low toxicity, strong biological activity, and ability to undergo diverse structural modifications. These compounds may directly kill the pathogens, but instead of enhancing the defense responses of plants, some of them may act as plant growth regulators [111,112]. In tomato plants, current research primarily focuses on detecting thiazoles, such as 2-isobutylthiazole, which is a well-established sulfur-containing volatile responsible for the characteristic green aroma of tomato fruit, and on elucidating the pathways by which they are formed [113]. Other analyses of fresh cultivars reported 2-propylthiazole and 2-s-butylthiazole as newly identified tomato sulfur volatiles, and found 2-s-butylthiazole to be aroma-active as well [18]. Moreover, benzothiazole and DMDS emission was shown to increase in the volatilome of tomato plant roots treated with bacterial and fungal inoculants, *Bacillus amyloliquefaciens*, *Pseudomonas azotoformans*, *Trichoderma harzianum*, and *Rhizophagus irregularis*, under leaf herbivory stress of insect *Spodoptera exigua* [114]. Antimicrobial functions of benzothiazole against different pathogens, including *Alternaria solani* and *B. cinerea*, were also presented by other researchers [115,116].

Thiophene and its derivatives are produced as part of the chemical defense mechanism in numerous plant species towards different pathogens, including nematodes, insects, fungi, bacteria and viruses. Their inhibitory effect against several soil-borne and foliar plant pathogens, including *Rhizoctonia solani*, *Sclerotinia sclerotiorum*, and *Sclertium rolfsii*, was presented. They act as repellents or toxic substances, or have anti-nutritional effects on herbivores [117]. Several thiophenes detected in tomato plants were presented previously as important volatile biomarkers of plant development or disease, including thiophen-2-ylmethanethiol emphasized as one of the important compounds playing a decisive role in aroma formation during apricot fruit development [107], 2-pentylthiophene in potato tubers inoculated with pectinolytic bacteria *Dickeya* spp. which causes soft rot [118], or 2-ethylthiophene which enhanced the emission from leaves and was detected in transgenic tomato plants with modified lipid metabolism, suggesting its involvement in the plant’s odor profile and potentially in plant defense [119].

Regarding other VSCs present in tomato, simple thioester S-methyl thioacetate (SMT) together with DMDS were shown to be co-linked to enhance germination, alter root architecture, and increase the leaf area and survival rates of *Arabidopsis thaliana* under salt stress conditions [120], and in terms of biocontrol, SMT exhibited significant fumigant toxicity against nematodes *Caenorhabditis elegans* and *Meloidogyne incognita* [121]. In turn, methionol was emphasized among compounds enhancing tomato seedling root growth under sterile conditions [122].

There is currently a steady increase in publications on the production and emission of endogenous VSCs by plants including tomato in changing environments. The implementation of modern technologies may help to discover these compounds in various crops, including tomato plants, and demonstrate that even though they are in the minority, these compounds can play a significant role in protecting plants from abiotic or biotic factors.

## 6. Conclusions and Future Perspectives

VSCs represent a chemically diverse and biologically important subgroup of plant volatile organic compounds whose significance extends far beyond their relatively low abundance. In tomato (*Solanum lycopersicum* L.), VSCs comprise sulfides, thiols, thiazoles, thiophenes, thioesters, and several low-molecular-weight sulfur-containing molecules that originate from sulfur assimilation pathways and the metabolism of Cys and Met. These compounds contribute substantially to aroma perception owing to their exceptionally low odor thresholds, but they also participate in a wide range of physiological and ecological processes, including stress adaptation, redox regulation, plant defense, and interactions with microorganisms, herbivores, and neighboring plants.

This review highlights the current understanding of importance of developing various techniques for detecting VSCs produced by tomato and other plants, which will enable us to gain a clearer understanding of their generation and emission. Attention is paid to determine possible VSC biosynthesis, occurrence, and biological functions in tomato plants. Particular attention is given to the close relationship between sulfur metabolism and VSC formation, as well as to the emerging evidence supporting their involvement in plant responses to both biotic and abiotic stresses. Although several sulfur-containing volatiles, including methanethiol, dimethyl sulfide, dimethyl disulfide, dimethyl trisulfide, thiazoles, and thiophenes, have been identified in tomato, many biosynthetic pathways remain incompletely resolved, and the physiological roles of numerous compounds are still poorly understood.

VSCs remain substantially underexplored, largely due to analytical limitations. Their low concentrations, high reactivity, susceptibility to oxidation, thermal instability, and often inadequate recovery by commonly applied untargeted volatilomic methodologies frequently result in their omission or underrepresentation in scientific studies. Consequently, the sulfur fraction of the plant volatilome is often treated with less attention than other volatile classes, despite its potentially disproportionate contribution to plant physiology and sensory properties. This analytical bias likely affects not only tomato research but also investigations plant volatilomes in general.

Future advances in sulfur-selective detection, optimized extraction strategies, and high-resolution chromatographic techniques are expected to reveal the considerably broader diversity and biological relevance of plant VSCs than currently recognized. Such analyses, supported by complementary analytical methods including microscopic, spectroscopic and immunological techniques, could enable improved detection of various sulfur compounds (such as VSCs) in plant material in the future, as well as monitoring changes in their profiles and emission intensities over time. Another crucial aspect of future research will be optimizing environmental sampling and analysis procedures to avoid interference from extraneous factors such as thermal processing, which significantly affects analytical results. In advancing modern VSC detection techniques, progress in other analytical fields, including theoretical chemistry and physics, is also essential. This facilitates a deeper understanding of these compounds’ properties and the development of data analysis tools based on machine learning algorithms and other AI-driven methods.

Significant knowledge gaps and the need for assumptions regarding sulfur metabolism and VSCs in tomatoes present researchers with substantial challenges, ranging from detecting metabolic changes in the precursor compounds that give rise to VSCs to analyzing the activity of enzymes involved in this metabolism and investigating the molecular basis of VSC emissions. Further research in this area could help to determine the following: (i) spatiotemporal changes in VSC precursors and VSC production across different tomato wild and genetically modified cultivars, organs, tissues and cellular organelles; (ii) VSC synthesis pathways at molecular and biochemical levels in plants under stable conditions, during flowering, ripening or senescence, and under various biotic and abiotic stresses; and (iii) interactions and interdependencies between VSC production and emission and that of other VOCs.

A more comprehensive characterization of VSCs belonging to different groups, including thiols, sulfides, thiazoles, and thiophenes, will be essential for understanding sulfur-dependent signaling networks, plant stress resilience, crop quality, and aroma formation in tomato and other agriculturally important species. In light of contemporary research trends, such an expansion of knowledge is poised to make a substantial contribution to a more profound comprehension of the fundamental interactions between plants—including tomatoes—and other organisms, such as other plants and microorganisms. This could result in the development of practical applications for integrated, eco-friendly crop and food protection.

## Figures and Tables

**Table 2:** Main VSCs detected in *S. lycopersicum* plant. The odor threshold values and aroma descriptions are based on The Good Scents Company Information System [46].

VSC Compound	Chemical Formula	Chemical Group	Aroma Description	Odor Threshold
Dimethyl sulfide	C_2_H_6_S	sulfides	Cabbage, sulfurous	0.1 ppm
Dimethyl disulfide	C_2_H_6_S_2_	sulfides	Garlic, sulfurous	0.2 ppm
Dimethyl trisulfide	C_2_H_6_S_3_	sulfides	Onion, meaty, sulfurous	<2 ppm
Ethyl isopropyl disulfide	C_5_H_12_S_2_	sulfides	Onion	n/d
2-Methyl-3-furanthiol	C_5_H_6_OS	thiols	Sulfury, meaty, fish, metallic	0.005 ppm
2-Methyl-1,3-dithiolane	C_4_H_8_S_2_	thiols	Smoky, roasted, onion	0.05 ppm
2-Propylthiazole	C_6_H_9_NS	thiazoles	Green, vine, herbal	n/d
2-Secbutylthiazole	C_7_H_11_NS	thiazoles	Green, vine, tomato	0.005 ppm
2-Isobutylthiazole	C_7_H_11_NS	thiazoles	Green, tomato leaf	0.002 ppm
Benzothiazole	C_7_H_5_NS	thiazoles	Vegetable, nutty, coffee	<3 ppm
2-Ethylthiazole	C_5_H_7_NS	thiazoles	Green, nutty	0.3 ppm
Thiazole	C_3_H_3_NS	thiazoles	Fishy, nutty, meaty	<5 ppm
2,4,5-Trimethylthiazole	C_6_H_9_NS	thiazoles	Vegetable, cocoa, coffee	1 ppm
4-Methyl-5-vinylthiazole	C_6_H_7_NS	thiazoles	Musty, nutty, vegetable	<5 ppm
2-Pentylthiophene	C_9_H_14_S	thiophenes	Fatty	n/d
2-Ethylthiophene	C_6_H_8_S	thiophenes	Styrene	0.01 ppm
Thiophen-2-ylmethanethiol	C_5_H_6_S_2_	thiophenes/ thiols	Roasted, coffee, fishy	<5 ppm
2-Acetylthiophene	C_6_H_6_OS	thiophenes	Onion, malty, roasted	0.2 ppm
Methional	C_4_H_8_OS	other	Cooked potato	0.01 ppm
S-Methyl thioacetate	C_3_H_6_OS	other (thioester)	Sulfurous, cheese, vegetable	n/d
S-Butan-2-yl 3-methylbutanethioate	C_9_H_18_OS	other (thioester)	Herbal, spicy	0.2 ppm
2-(Methylthio)ethanol	C_3_H_8_OS	other	Sulfurous, meaty	n/d
Methionol	C_4_H_10_OS	other	Sulfurous, onion, sweet, soup	0.01 ppm

## Data Availability

No new data were created or analyzed in this study. Data sharing is not applicable to this article.
